# The Interaction of DMRTA2 with HSP90β Inhibits p53 Ubiquitination and Activates the p53 Pathway to Suppress the Malignant Progression of Non-Small-Cell Lung Cancer

**DOI:** 10.3390/cimb47070497

**Published:** 2025-06-28

**Authors:** Shiyang Deng, Ling Li, Jiang Du

**Affiliations:** Department of Pathology, The First Affiliated Hospital of China Medical University, 155 Nanjing Street, Heping District, Shenyang 110001, China; dengshiyang@cmu.edu.cn (S.D.); zhangh@cmu.edu.cn (L.L.)

**Keywords:** *DMRTA2*, *p53*, HSP90β, non-small-cell lung cancer, proliferation

## Abstract

**Background:** Lung cancer, predominantly NSCLC (80%), has a poor prognosis due to late diagnosis and limited treatment efficacy. *DMRTA2 (DMRT5)*, a transcription factor linked to neural/germ cell development, is overexpressed in NSCLC per TCGA data, indicating its potential role in tumorigenesis and as a therapeutic target. **Methods:** Conduct a comprehensive search of the relevant theoretical foundations. Based on this, differential expression analysis will be performed using the DESeq2 package in R on RNA-seq data from lung adenocarcinoma and lung squamous cell carcinoma in the TCGA database. The research will then employ various methods, including CRISPR genome editing, MTS assay, flow cytometry, Western blot, co-immunoprecipitation, immunofluorescence, and qRT-PCR. **Results:** Through experimental validation, we found that *DMRTA2* mRNA is highly expressed in non-small-cell lung cancer (NSCLC) tissues and is negatively correlated with poor prognosis. *DMRTA2* binds to HSP90β, inhibiting the interaction between HSP90β and *p53*, thereby suppressing *p53* ubiquitination and nuclear export. This activates the *p53* pathway, inhibiting the proliferation and invasion of lung cancer cells. **Conclusions:** In NSCLC, *DMRTA2* acts as a context-dependent regulator, stabilizing wild-type *p53* through competitive HSP90β binding to suppress tumors, while in p53-compromised cells, potentially engaging HSP90β or alternative pathways to promote malignancy. Its dual localization and transport interactions reveal multifunctional, stress-responsive roles beyond transcription.

## 1. Introduction

Lung cancer is a complex, multi-step disease with various histological subtypes, and is a leading cause of cancer-related deaths in China. In recent years, both the incidence and mortality rates of lung cancer have sharply risen, driven by factors such as smoking, air pollution, and occupational exposure [1,2,3]. Histologically, lung cancer is classified into two main categories: non-small-cell lung cancer (NSCLC) and small-cell lung cancer (SCLC). NSCLC [4,5], which comprises adenocarcinoma, squamous cell carcinoma, and large cell carcinoma, accounts for approximately 80% of all lung cancer cases. Most NSCLC patients are diagnosed at advanced stages, where treatment options are limited, resulting in a five-year survival rate of less than 20% [5,6,7]. Currently, the main treatment modalities for advanced lung cancer are chemotherapy and radiotherapy [8,9]; however, drug resistance to chemotherapy and resistance to radiotherapy have become major obstacles in the treatment of lung cancer.

The *TP53* gene is located at 17p13.1 in humans and encodes the *p53* protein, which consists of 393 amino acids with a molecular weight of approximately 53 kDa. p53 is an essential transcription factor that contains a transcriptional activation domain at its N-terminus, a DNA-binding domain (DBD) in the central region, and a nuclear localization sequence along with a tetramerization domain at its C-terminus [10,11,12]. As the most common tumor suppressor, *p53* inhibits tumor cell proliferation by inducing cell cycle arrest and promoting apoptosis [13,14]. The half-life of wild-type *p53* is only 15–30 min. Upon DNA damage, oncogene activation, loss of cell contact, or hypoxia, *p53* is activated to regulate the transcription of downstream genes and halt cell proliferation [15,16,17]. The inactivation of *p53* promotes cancer development [18]. The main mechanisms include mutations in the *TP53* gene or post-translational modifications and degradation of the *p53* protein [19]. *TP53* mutations may lead to a loss of *p53* function or the acquisition of new functions by mutant *p53*. The primary reason for the inactivation of wild-type *p53* is MDM2-mediated ubiquitination. *MDM2* acts as a negative regulator of *p53*, degrading it via the ubiquitin–proteasome pathway [20]. *MDM2* cooperates with the E2 ubiquitin-conjugating enzyme UBC5 to ubiquitinate *p53* at six lysine residues in the C-terminus (K370, K372, K373, K381, K382, K386) [21], promoting its degradation or nuclear export. Low levels of *MDM2* induce mono-ubiquitination, leading to the nuclear export of *p53*, whereas high levels induce poly-ubiquitination, promoting the nuclear degradation of *p53*. Studies have shown that *p53* degradation can occur in both the nucleus and cytoplasm, and the nucleocytoplasmic shuttling of *p53* and its co-localization with *MDM2* are crucial for its degradation process [22,23,24].

Heat shock protein 90 (HSP90) is an important molecular chaperone responsible for assisting the folding, maturation, and stabilization of a large number of client proteins. HSP90β is a cytoplasmic isoform of HSP90, consisting of 724 amino acids and containing three conserved domains: the N-terminal domain, the C-terminal domain, and the middle domain. The N-terminal domain possesses ATP-binding activity, which is crucial for the conformational changes of HSP90β. In the cytoplasm, HSP90β typically exists as a homodimer, cycling between closed and open conformations through ATP binding and hydrolysis, thereby facilitating the proper folding of client proteins or participating in other processes [25].

The *DMRTA2* gene is located at 1p32 and encodes the protein *DMRTA2*, a member of the DMRT family, which contains a DM domain that binds DNA and a DMA domain of yet an undefined function. The DM domain is a novel zinc finger structure composed of an α-helix and two antiparallel β-strands, with the α-recognition helix at the carboxy terminus capable of specifically recognizing DNA sequences and binding in the minor groove of the DNA double helix without inducing DNA bending [26]. Studies have shown that *DMRTA2*, as a transcription factor, participates in various biological processes, including the maintenance of neural progenitor cells [27], forebrain neurogenesis [28], and the development of germ cells [29,30,31,32]. However, the role of *DMRTA2* in cancer remains unclear, particularly regarding its expression pattern and biological function in lung cancer. To clarify the function of *DMRTA2* in NSCLC, we employed bioinformatics tools combined with a series of cell experiments to preliminarily investigate the impact of DMRTA2 on the malignant phenotype of NSCLC and its potential molecular mechanisms.

## 2. Methods and Materials

### 2.1. Bioinformatics

RNA sequencing data for lung adenocarcinoma and lung squamous cell carcinoma were obtained from The Cancer Genome Atlas (TCGA) database, and differential gene expression analysis was performed using the R (4.4.3) package DESeq2. The Gene Expression Omnibus (GEO) database was used to acquire the GSE176348 dataset, and differential analysis was conducted using the GEO2R online tool. The BioGRID ORCs database provided two CRISPR screen datasets related to cell proliferation: 209-PMID29083409 and 334-PMID29083409. The TIMER2.0 database was utilized for the expression analysis of *DMRTA2* across various cancers.

### 2.2. Cell Culture

Human bronchial epithelial (HBE) cells and non-small-cell lung cancer cell lines H1975, HCC827, LK2, A549, SK-MES-1, and H1299 were obtained from the American Type Culture Collection (ATCC, Manassas, VA, USA), while the human large-cell lung cancer cell line H460 was purchased from the Shanghai Cell Bank, Shanghai, China. H1299, A549, H1975, and HCC827 cells were cultured in RPIM-1640 medium containing 10% fetal bovine serum (FBS) and 1% penicillin–streptomycin mixed antibiotics. HBE, LK2, and SK-MES-1 cells were cultured in high-glucose DMEM medium with 10% FBS and 1% antibiotics (a mixture of penicillin and streptomycin). All cells were maintained in a 37 °C incubator with 5% CO_2_ and humidity.

### 2.3. Cell Transfection

The *pCMV6-Flag-DMRTA2* plasmid (EX-H2136-M35) and the control empty *pCMV6-Flag* plasmid were purchased from GeneCopoeia. PLentiCRISPRV2-U6-*DMRTA2* (human)-sgRNA-1~2-EF1a-Cas9-P2A-GFP-puro and PLentiCRISPRV2-GFP were obtained from Miaoling Biotechnology, Wuhan, China. Transfection was performed using Lipo8000™ (Beyotime Biotechnology Co., Ltd., Shanghai, China) according to the manufacturer’s instructions when the cell density reached 70–80%. After 6 h, fresh culture medium was added, and subsequent experiments were conducted 24–48 h post-transfection. All transfections were compared against the control group transfected with the empty plasmid.

### 2.4. CRISPR Genome Editing

Using the online tool CHOPCHOP (http://chopchop.cbu.uib.no/, accessed on 6 April 2024), we designed the knockout sequences for the *DMRTA2* gene. The selected sgRNA sequences were ACGCGTGTCAAGATATCCAGCGC and CTAAAGGCGTAGTCCATGGGTGG. These two sgRNA sequences were then separately cloned into the PLentiCRISPRV2-GFP vector.

### 2.5. MTS Experiment

After 24 h of transfection, digest the cells with 0.25% trypsin and resuspend them in 1 mL of complete culture medium. Collect the cell suspension into a 1.5 mL centrifuge. Gently pipette to mix the cell suspension, then take 10 μL of the cell suspension and add it to a centrifuge tube containing 90 μL of PBS for dilution. Wipe the cell counting plate and coverslip with alcohol. After mixing the diluted cell suspension, add 10 μL of the dilution to each side of the counting plate and count under a microscope. Based on the average count, calculate the total volume of the cell suspension and culture medium needed to seed 3000 cells per well in a 96-well plate. Incubate the cells in a 37 °C, 5% CO_2_ incubator for 6 h, then add 20 μL of MTS solution to each well. Continue incubating in the dark for 2–4 h and measure the absorbance at 490 nm using a microplate reader to obtain the data for day 1. The same method was used to measure absorbance on days 2–5 at the same time points. The obtained data were statistically analyzed using GraphPad Prism 10.2.1 software, with each experimental group repeated more than three times.

### 2.6. Colony Formation Experiment

Cells transfected for 24–48 h were digested with 0.25% trypsin to prepare a 1 mL cell suspension for counting. In a six-well plate, 2 mL of complete culture medium was added, followed by the addition of 1000 cells, which were gently mixed. The cells were then incubated at 37 °C with 5% CO_2_ for 7–14 days, with medium changes during this period until visible colonies formed. The cells were washed three times with PBS, and 1 mL of ice-cold methanol was added to each well and fixed at −20 °C for 15–20 min. After removing the methanol, the cells were washed three times with PBS, and 1 mL of crystal violet staining solution was added for 15–30 min, followed by rinsing with tap water. After drying the six-well plate, images were captured using an ECL bioimaging system, processed with ImageJ 1.8.0, and statistically analyzed using GraphPad Prism software. Each experimental group was repeated more than three times.

### 2.7. Transwell Experiment

Cells transfected for 24–48 h were digested with 0.25% trypsin to prepare a 1 mL cell suspension for counting. Each chamber was filled with 20,000 cells, with the lower chamber containing 20% FBS medium and the upper chamber containing 2% FBS medium. After incubating the cells at 37 °C with 5% CO_2_ for 24–48 h, they were washed three times with PBS. The cells were then fixed with ice-cold methanol for 15–20 min, washed three times with PBS, and stained with crystal violet for 15–30 min, followed by rinsing with tap water. Using a moist cotton swab, the cells on the inner side of the chamber were wiped away, and after drying, images were captured under an inverted microscope. Statistical analysis was performed using GraphPad Prism software, with each experimental group repeated more than three times.

### 2.8. Flow Cytometry

Following 24–48h transfection, cells were digested with 0.25% trypsin and transferred to 1.5 mL centrifuge tubes. After centrifugation at 1000 rpm for 5 min, the supernatant was discarded. Cells were washed with PBS, centrifuged at 2000 rpm for 5 min, and the supernatant removed. Ice-cold 70% ethanol was added for fixation (2 h overnight at 4 °C). After PBS washing, 500 μL PI/RNaseA staining solution was added per tube. Samples were protected from light for 30–60 min of room-temperature incubation before cell cycle analysis using a BD FACSCalibur flow cytometer (BD Biosciences, San Jose, CA, USA). All experiments included ≥3 biological replicates.

### 2.9. Western Blot Experiment

Lyse the cell pellet with NP40 lysis buffer containing 1% PMSF and phosphatase inhibitors on ice for 30 min, vortexing for 10 s every 10 min. After lysis, centrifuge at 12,000 rpm for 20 min at 4 °C. Collect the supernatant. Quantify the protein using the BCA method, then add SDS-PAGE loading buffer to prepare equal amounts of protein samples. Denature the samples at 95 °C for 5–7 min. The protein samples can be directly used for Western blot experiments or stored at −80 °C. Prepare 7.5%, 10%, and 15% SDS-PAGE gels and run the electrophoresis for 50–70 min, monitoring the position of the samples. After electrophoresis, transfer at a constant voltage of 100 V for 70 min (adjustable). After transfer, block the PVDF membrane in 5% non-fat milk for 2 h. Wash with TBST, then incubate with the primary antibody overnight. The next day, wash with TBST again and incubate with HRP-conjugated secondary antibody for 2 h. Finally, use ECL detection reagents for imaging.

### 2.10. Immunoprecipitation Experiment

A portion from within the protein extract was used as INPUT, added to SDS-PAGE protein upload buffer, heated and stored at −80 °C. After removing the non-specific binding using Protein A + GAgarose (Beyotime, Shanghai, China), centrifuge at 4 °C, 1500 rpm for 5 min, and take the supernatant to a new centrifuge tube. The target antibody and anti-mouse/rabbit IgG (Beyotime) were added separately, and then another 50 μL of Protein A + G Agarose was added to each tube and incubated overnight at 4 °C on a shaker. On the next day, the agarose beads were washed three times with NP40 buffer containing 1% PMSF and phosphatase inhibitor, SDS-PAGE protein uploading buffer was added, and the samples were cooked at 100 °C for 5–10 min, and finally, the immunoblotting experiments were carried out.

### 2.11. Immunofluorescence

Cells were digested with 0.25% trypsin and spread on cell crawlers for overnight incubation. On the next day, the medium was removed and washed with PBS, then 500 uL of 4% paraformaldehyde was added to each well and fixed for 15 min. After fixation, the wells were washed with PBS, punched with 0.1% Triton for 10–15 min, washed with PBS for three times, and closed in PBS with 3% BSA for 2 h. The cells were incubated with a 1:50 dilution of DMRTA2 Rabbit Antibody overnight at 4 °C. The next day, the cells were washed three times with PBS, diluted at 1:200 in Goat Anti-Rabbit Antibody. On the next day, PBS was washed three times, and a 1:200 dilution of Goat Anti-Rabbit IgG (H + L) was incubated away from light for 1.5–2 h. After PBS washing, the nuclei were stained with DAPI for 10 min, and after washing with PBS three times, the slices were sealed with anti-fluorescence quenching sealer and visualized for imaging in a confocal microscope.

### 2.12. qRT-PCR

The collected cell precipitates were lysed and total RNA was extracted, and the quality and concentration of RNA were detected by a microspectrophotometer. Reverse transcription was performed and qPCR experiments were carried out.

### 2.13. Statistical Analysis

All experimental data were statistically analyzed using GraphPad Prism 10.2.1 and SPSS24.0 software, and the results are expressed as mean ± standard deviation (x ± s), and each test was repeated at least three times. Statistical analyses were performed using Student’s test or ANOVA, and the χ^2^ test was used to assess the correlation between clinicopathological factors and *DMRTA2* expression. Differences were considered statistically significant when *p* < 0.05.

## 3. Results

### 3.1. DMRTA2 Is Highly Expressed in NSCLC and Negatively Associated with Poor Prognosis

**Figure 1:**
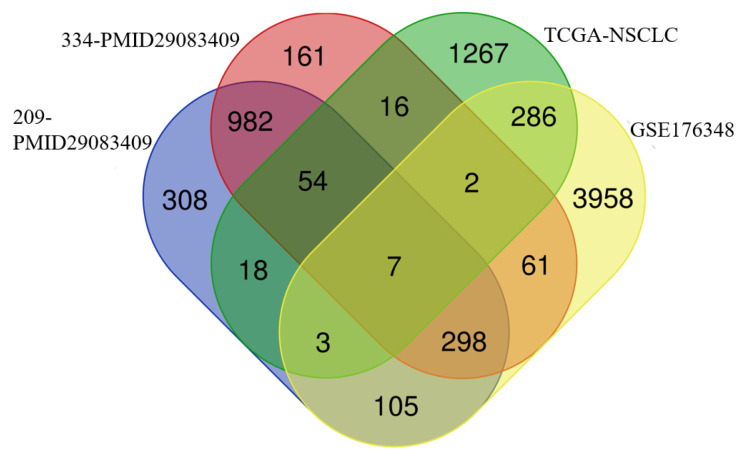
The intersection of four datasets was used to obtain the core genes.

To identify genes associated with NSCLC proliferation, we intersected differentially expressed genes from the TCGA database for lung adenocarcinoma and squamous cell carcinoma, as well as those from the GSE176348 dataset and the CRISPR screens dataset (209-PMID29083409 and 334-PMID29083409), resulting in seven core genes. Among these, *DMRTA2*, which has not been studied in lung cancer, captured our attention (Figure 1).

**Figure 2:**
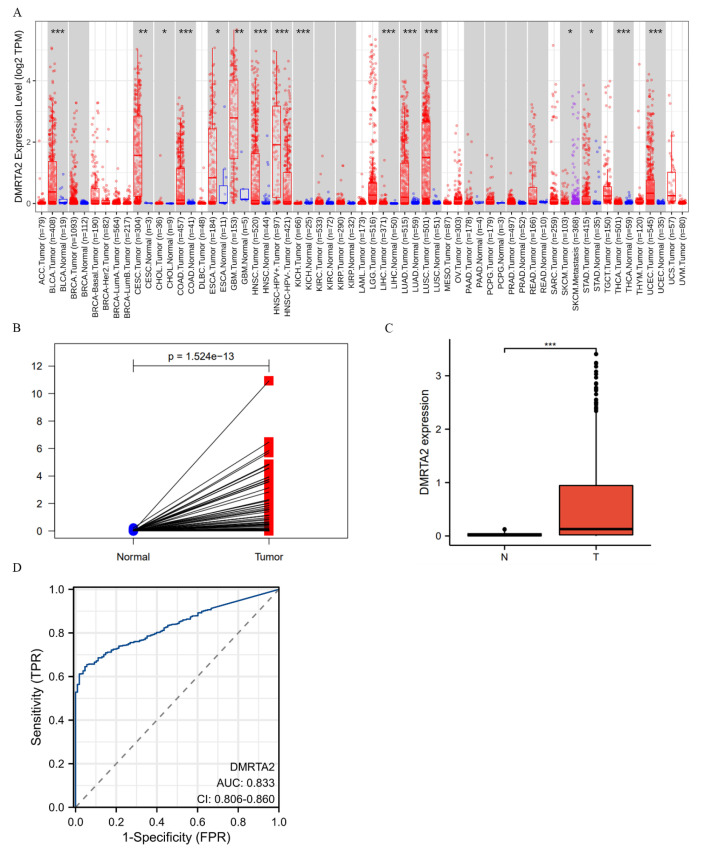
Expression of *DMRTA2* in lung cancer and adjacent normal lung tissues. (**A**) The mRNA level of *DMRTA2* in various cancer tissues is higher than that in para-cancer tissues. (**B**) The expression of *DMRTA2* in unmatched lung cancer and adjacent tissues was detected by the TCGA database, and the mRNA level of *DMRTA2* was highly expressed in lung cancer tissues. (**C**) The mRNA expression of *DMRTA* in paired lung cancer and adjacent tissues was detected by the TCGA database, and *DMRTA2* was highly expressed in paired lung cancer tissues. (**D**) The ROC curve of *DMRTA2* in NSCLC was plotted using the TCGA database, and the AUC was 0.833 (95%CI, 0.806–0.860). *p* < 0.05 is statistically significant; * *p* < 0.05, ** *p* < 0.01, and *** *p* < 0.001.

**Figure 3:**
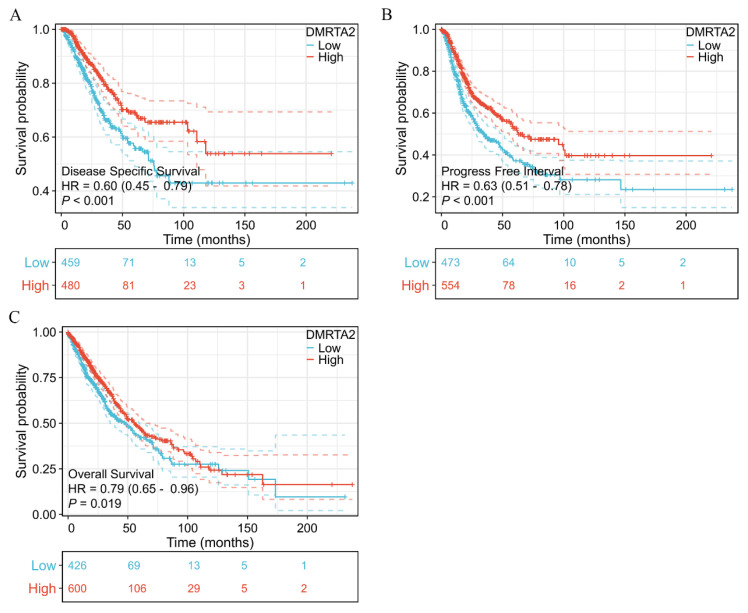
Kaplan–Meier prognostic analysis of *DMRTA2* in NSCLC. (**A**) Disease-specific survival. (**B**) Progression-free survival. (**C**) Overall survival. *p* < 0.05 was statistically significant.

**Figure 4:**
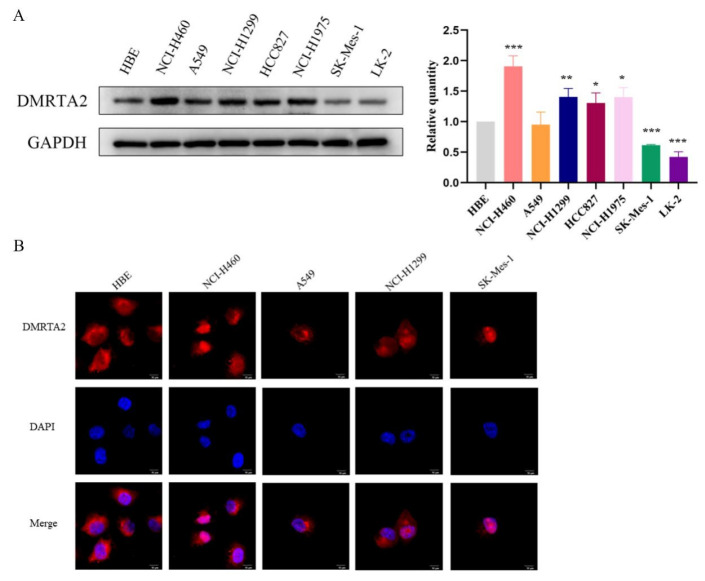
Protein expression and localization of *DMRTA2* in lung cancer cell lines. (**A**) Western blot showed that *DMRTA2* was highly expressed in H460, H1299, HCC827, and H1975 cells, and had a low expression in SK-MES-1 and LK-2 cells. (**B**) Immunofluorescence results show that *DMRTA2* is mainly expressed in the cytoplasm and nucleus. *p* < 0.05 was statistically significant; * *p* < 0.05, ** *p* < 0.01, and *** *p* < 0.001. scale bar: 20 μm.

We assessed the expression and characteristics of *DMRTA2* across various databases. TIMER2.0 results indicate that *DMRTA2* is highly expressed in lung adenocarcinoma, lung squamous carcinoma, liver cancer, and colon cancer (Figure 2A). TCGA data reveal that its mRNA levels are elevated in paired and non-paired lung cancer tissues compared to adjacent normal tissues (Figure 2B,C). ROC curve analysis demonstrated the potential of *DMRTA2* to discriminate lung cancer, with an AUC of 0.833 (Figure 2D). Kaplan–Meier analysis showed that high *DMRTA2* expression is associated with better survival rates (Figure 3A–C). Additionally, *DMRTA2* expression correlated with T stage, smoking history, and TP53 expression. Western blot analysis indicated that *DMRTA2* levels are higher in H1299, HCC827, H1975, and H460 cells compared to normal lung cells (HBEs), while lower in SK-MES-1 and LK2 cells (Figure 4A). Immunofluorescence experiments demonstrated its localization in the nucleus and cytoplasm, with some cells showing nucleolar localization (Figure 4B).

### 3.2. DMRTA2 Affects the Malignant Phenotype of Lung Cancer Cells

**Figure 5:**
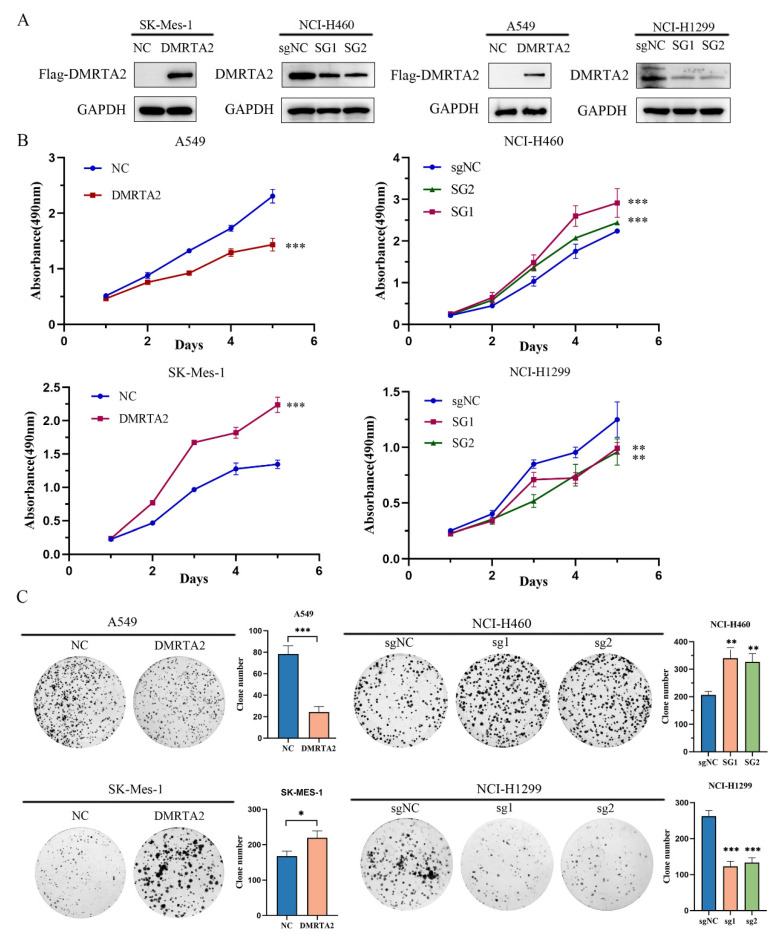
Effect of *DMRTA2* expression on the proliferation of NSCLC cells. (**A**) Detection of *DMRTA2* overexpression in A549 and SK-MES-1 cells and knockout efficiency in H1299 and H460 cells. (**B**) MTS assay was used to detect cell proliferation after the overexpression of *DMRTA2* in A549 and SK-MES-1 cells, and after the knockout of *DMRTA2* in H1299 and H460 cells. (**C**) Colony formation assay was performed to detect the colony formation ability of cells after the overexpression of *DMRTA2* in A549 and SK-MES-1 cells, and after the knockout of *DMRTA2* in H1299 and H460 cells. * *p* < 0.05, ** *p* < 0.01, and *** *p* < 0.001.

**Figure 6:**
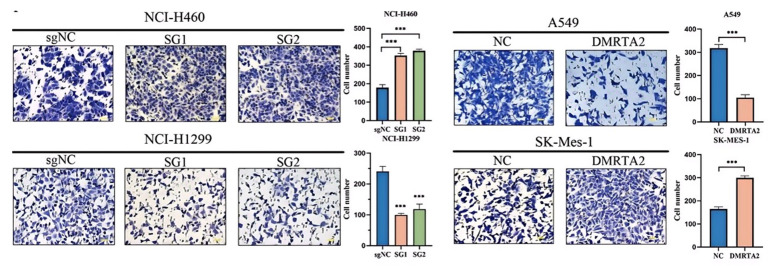
Effect of *DMRTA2* expression on the invasion ability of NSCLC cells. Transwell assay detected the invasion ability of cells after the overexpression and knockout of *DMRTA2* in A549, SK-MES-1, H1299, and H460 cells. *** *p* < 0.001. scale bar: 50 μm.

To investigate the function of *DMRTA2* in lung cancer, we overexpressed *DMRTA2* in SK-MES-1 and A549 cells, while knocking it out in H460 and H1299 cells (Figure 5A). We evaluated its effects on proliferation and invasion using MTS assays (Figure 5B), colony formation assays (Figure 5C), and Transwell experiments (Figure 6). The results indicate that *DMRTA2* inhibits cell proliferation and invasion in A549 and H460 cells, whereas it promotes these functions in SK-MES-1 and H1299 cells. GSEA analysis suggested that *DMRTA2* is associated with cell cycle pathways, and flow cytometry and Western blot results confirm that *DMRTA2* has distinct regulatory effects on the cell cycle across different cell lines. These findings suggest that *DMRTA2* plays a significant role in lung cancer progression.

### 3.3. DMRTA2 Inhibits Lung Cancer Cell Proliferation and Invasion by Regulating the p53 Signaling Pathway

**Figure 7:**
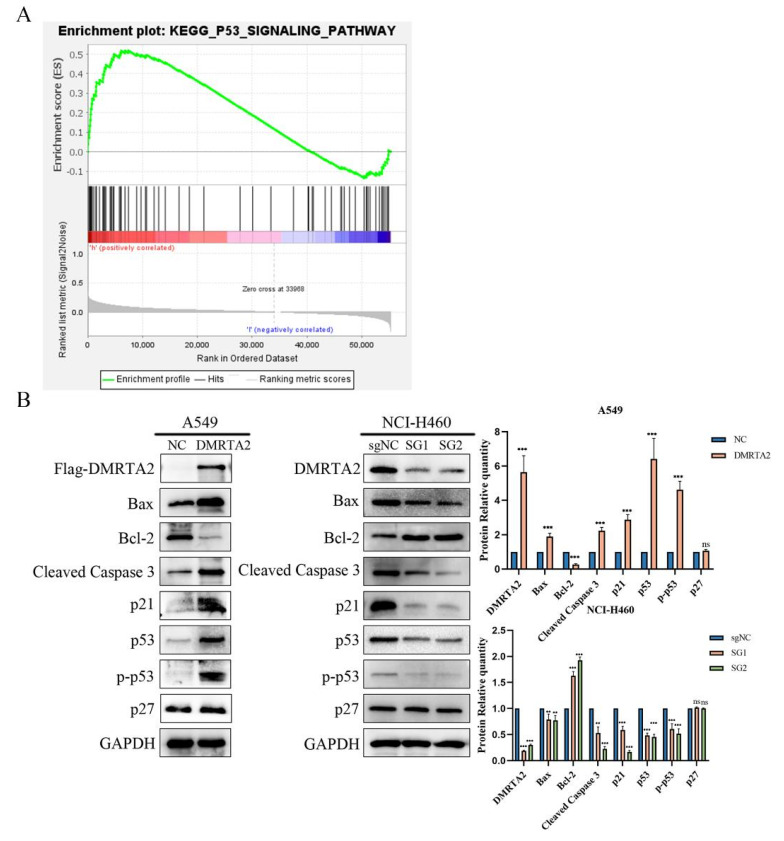
*DMRTA2* inhibits the malignant phenotype of *p53* wild-type lung cancer cells by regulating the *p53* pathway. (**A**) GSEA enrichment analysis showed that the expression of *DMRTA2* was correlated with the *p53* pathway (FDR q-value= 0.018, *p* < 0.05). (**B**) Western blot analysis of the effects of overexpression of *DMRTA2* in A549 and the knockout of *DMRTA2* in H460 on *p53* pathway-associated proteins. *p* < 0.05 was statistically significant; ** *p* < 0.01, and *** *p* < 0.001, ns means not significant.

**Figure 8:**
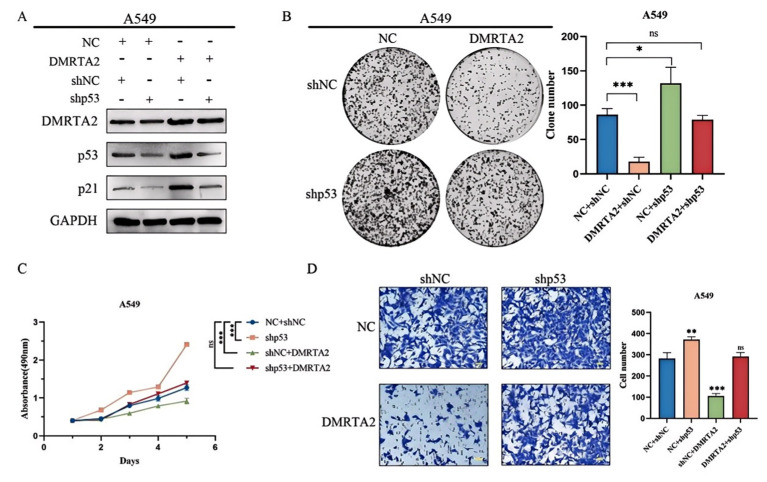
*DMRTA2* inhibits the *p53* malignant phenotype of wild-type lung cancer cells by regulating the *p53* pathway. (**A**) Effects of the overexpression of *DMRTA2* on *p53* and *p21* proteins after *p53* knockdown by Western blot analysis. (**B**) Colony formation experiment was conducted to detect the effect of the overexpression of *DMRTA2* on cell clonogenesis after *p53* knockdown. (**C**) The effect of the overexpression of *DMRTA2* on cell proliferation after the p53 knockdown was detected by MTS assay. (**D**) Effect of the overexpression of *DMRTA2* on cell invasion ability after *p53* knockout. *p* < 0.05 was statistically significant; * *p* < 0.05, ** *p* < 0.01, and *** *p* < 0.001, ns means not significant. scale bar: 50 μm.

**Figure 9:**
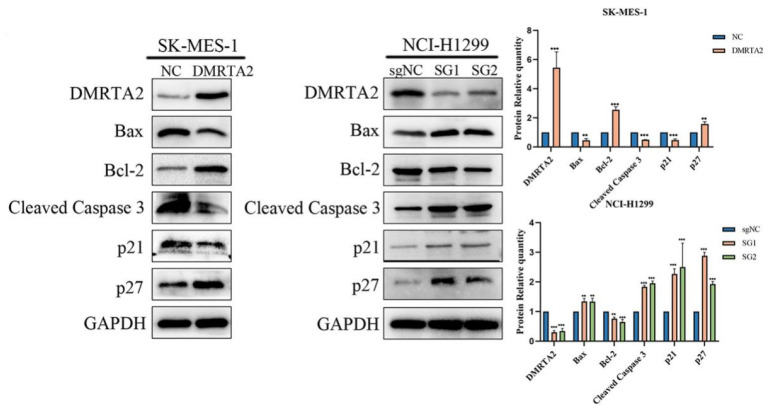
Effect of *DMRTA2* on downstream proteins of the *p53* pathway in *p53* mutant and *p53-deficient* cells. Western blot analysis of the effects of the overexpression of *DMRTA2* in SK-MES-1 and the knockout of *DMRTA2* in H1299 on *p53* pathway-related proteins. *p* < 0.05 was statistically significant; ** *p* < 0.01, and *** *p* < 0.001.

To investigate the reasons for the differential expression of *DMRTA2* in various cell lines, we first considered the genotypic differences among the cell lines. According to the Cellosaurus database, both A549 and H460 cells are p53 wild-type cells with KRAS and STK11 mutations, while SK-MES-1 has a p53 mutation, and H1299 is p53-null with an NRAS mutation. GSEA analysis indicated that *DMRTA2* is associated with the *p53* pathway (Figure 7A). Western blot analysis revealed that in A549 cells, the overexpression of *DMRTA2* upregulated *p53* and its downstream proteins (such as p21, BAX, Cleaved Caspase 3, etc.) while inhibiting Bcl-2. In contrast, in H460 cells, the knockout of *DMRTA2* yielded opposite results (Figure 7B). Further functional experiments demonstrated that the knockdown of *p53* could reverse the inhibitory effect of *DMRTA2* on the proliferation and invasion of A549 cells, suggesting that *DMRTA2* suppresses tumor malignant phenotypes through the *p53* pathway in *p53* wild-type cells (Figure 8A–D). However, in *p53* mutant or null SK-MES-1 and H1299 cells, the trends of downstream protein changes in the *p53* pathway were inconsistent (Figure 9), indicating the possibility of other mechanisms regulating the function of *DMRTA2*.

### 3.4. DMRTA2 Inhibits p53 Ubiquitination and Affects p53 Entry and Exit from the Nucleus

**Figure 10:**
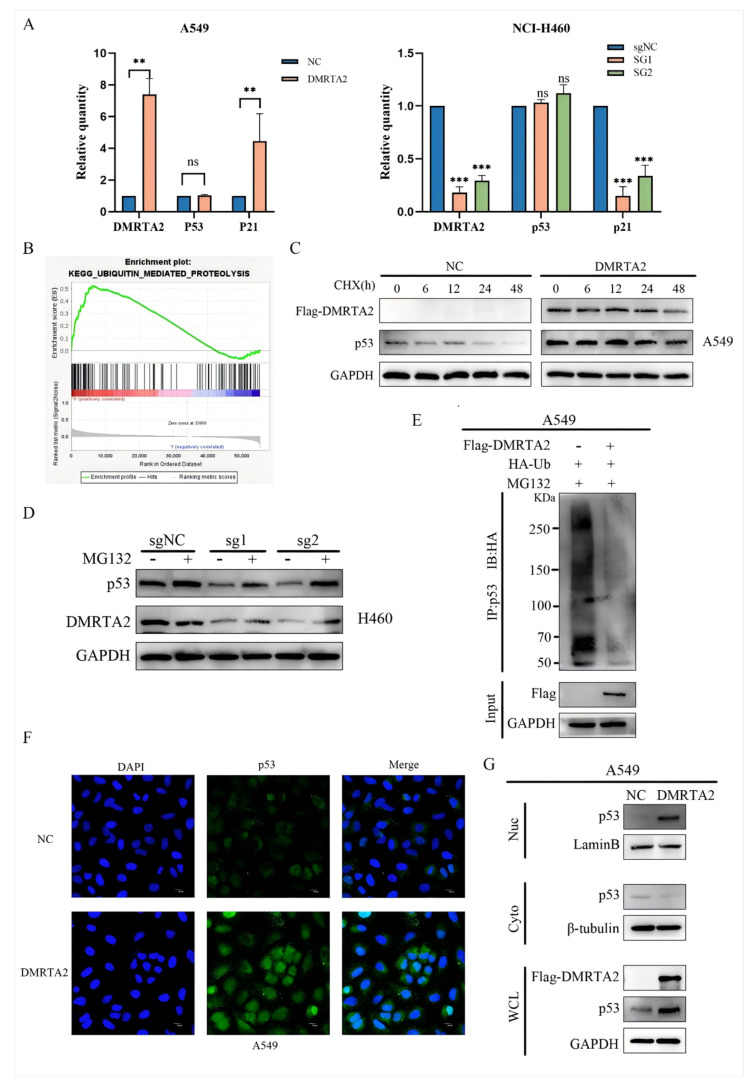
*DMRTA* affects the stability of the *p53* protein and inhibits *p53* ubiquitination. (**A**) qRT-PCR assay was used to detect the effect of the overexpression or *DMRTA2* knockout on *p53* transcription level. (**B**) GSEA results show that *DMRTA2* is associated with ubiquitin-mediated protein degradation (FDR q-value= 0.007, *p* < 0.05). (**C**) A549 cells were cultured for different times after adding actinomycosterone, and the influence of the overexpression of *DMRTA2* on the stability of *p53* protein was detected by Western blot. (**D**) MG132 was added into H460 cells, and the effect of the *DMRTA2* knockout on the proteasome degradation pathway of *p53* was detected by Western blot. (**E**) A549 cells were simultaneously transfected with *DMRTA2* plasmid and Ub plasmid to detect the effect of the overexpression of *DMRTA2* on the degree of *p53* ubiquitination. (**F**) The effect of the overexpression of *DMRTA2* on the subcellular localization of *p53* was detected by immunofluorescence assay. (**G**) After the overexpression of *DMRTA2* in A549 cells, a nuclear plasma separation experiment was conducted, and the influence of the overexpression of *DMRTA2* on the entry and exit of *p53* in the nucleus was detected by Western blot. *p* < 0.05 was statistically significant; ** *p* < 0.01, and *** *p* < 0.001, ns means not significant. scale bar: 10 μm.

The results indicate that *DMRTA2* can regulate wild-type *p53* protein levels, but it remains unclear whether this occurs at the mRNA level. Since *DMRTA2* is a transcription factor, qPCR analysis revealed that *DMRTA2* does not affect *p53* transcription but significantly regulates the mRNA expression of its target gene, *p21* (Figure 10A). GSEA analysis suggested a link between *DMRTA2* and ubiquitin-mediated protein degradation (Figure 10B), prompting us to investigate *p53* protein stability. CHX experiments demonstrated that *p53* degradation was slowed in A549 cells overexpressing *DMRTA2* (Figure 10C). Additionally, treatment with MG132 in H460 cells reversed the downregulation of p53 caused by *DMRTA2* knockout (Figure 10D). Further experiments indicated that *DMRTA2* inhibits *p53* ubiquitination (Figure 10E), and immunofluorescence and nuclear–cytoplasmic separation assays showed an increase in nuclear *p53* levels following *DMRTA2* overexpression (Figure 10F,G), suggesting that *DMRTA2* influences *p53* stability and subcellular localization.

### 3.5. DMRTA2 Binding to HSP90β Inhibits p53 Degradation

**Figure 11:**
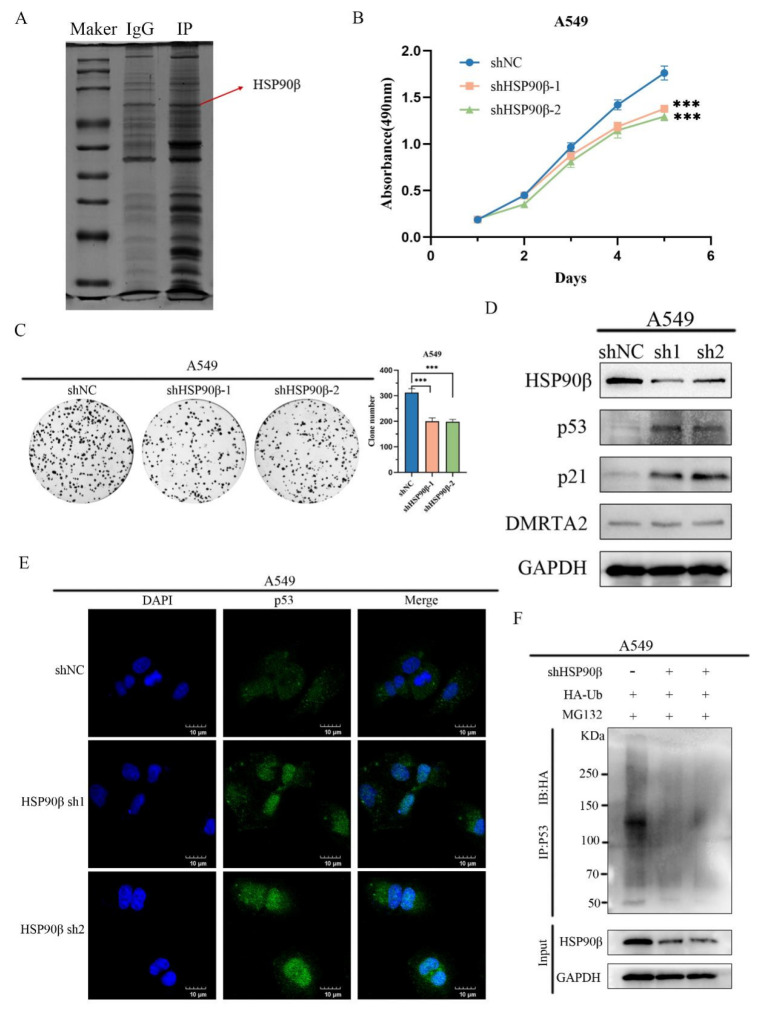
Knockdown of HSP90β, inhibition of *p53* ubiquitination, and inhibition of lung cancer cell proliferation. (**A**) Protein mass spectrometry for proteins that may interact with *DMRTA2*. (**B**) MTS assay was used to detect the effect of HSP90β knockdown on cell proliferation in A549. (**C**) Colony formation assay to detect the effect of knocking down HSP90β in A549 on cell clonogenesis. (**D**) Western blot analysis of the effect of knocking down HSP90β on *p53* protein expression in A549. (**E**) The effect of HSP90β knockdown on *p53* subcellular localization was detected by immunofluorescence assay. (**F**) Western blot analysis of the effect of knocking down HSP90β on *p53* ubiquitination. *p* < 0.05 was statistically significant; *** *p* < 0.001.

**Figure 12:**
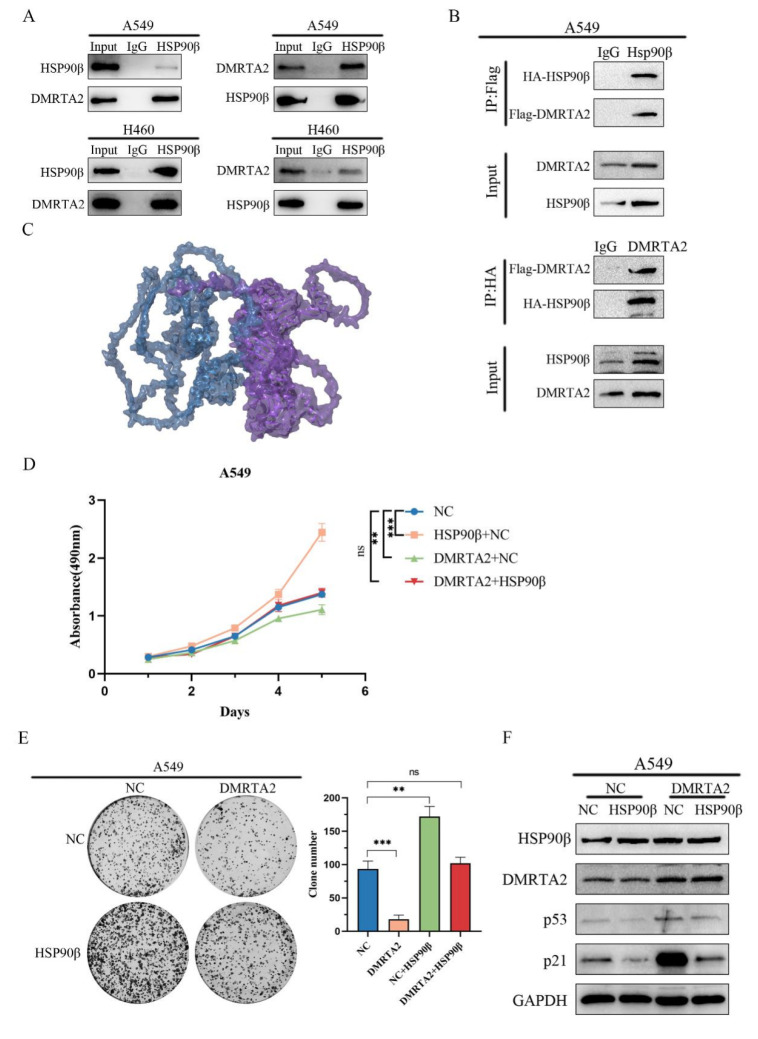
Binding of *DMRTA2* to HSP90β and inhibition of *p53* degradation. (**A**) Exogenous immunoprecipitation experiment verified the binding of *DMRTA2* to HSP90β. (**B**) Computer simulations of protein–protein interactions show that *DMRTA2* binds stably to HSP90β. (**C**) Endogenous immunoprecipitation assay verified the binding of *DMRTA2* and HSP90β. (**D**) MTS assay examined the effect of the overexpression of *DMRTA2* on cell proliferation after the overexpression of HSP90β in A549 cells. (**E**) Colony formation assay to determine the effect of the overexpression of *DMRTA2* on cell clonogenesis after the overexpression of HSP90β in A549 cells. (**F**) Western blot analysis of the effect of the overexpression of *DMRTA2* on *p53* protein expression after the overexpression of HSP90β in A549 cells. *p* < 0.05 was statistically significant; ** *p* < 0.01, and *** *p* < 0.001, ns means not significant.

**Figure 13:**
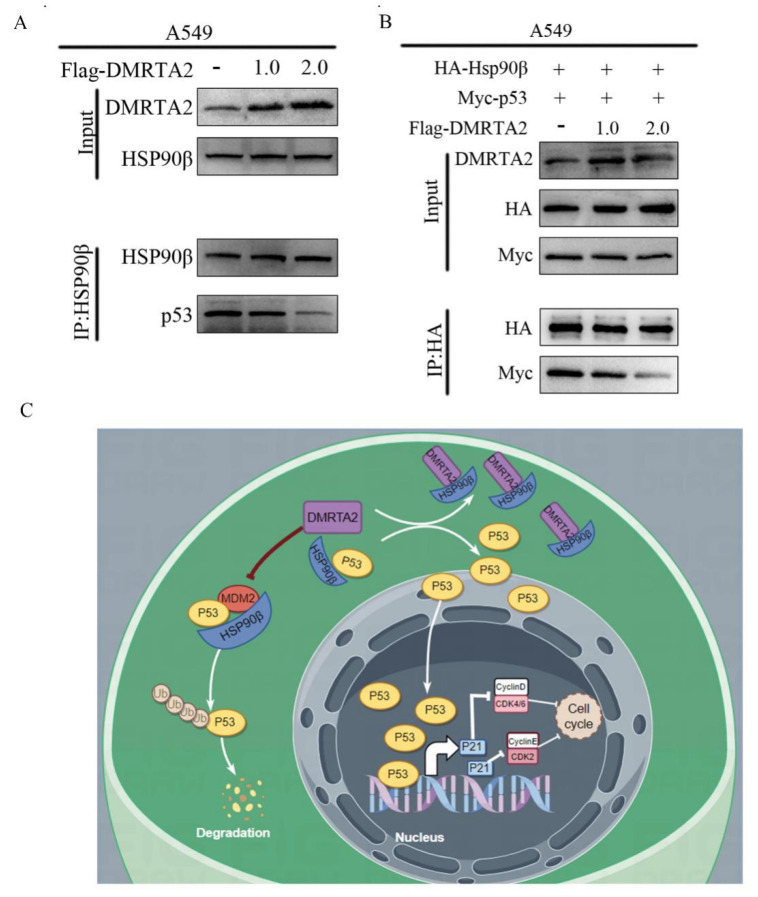
DMRTA2 binds to HSP90β and competitively inhibits the binding of p53 to HSP90β. (**A**) Gradient overexpression of DMRTA2 in A549 was performed to detect changes in p53 bound to HSP90β. (**B**) HSP90β and p53 were co-transfected in A549 cells at the same time, and gradient overexpression of DMRTA2 was performed to detect the changes in p53 bound to HSP90β. (**C**) Schematic diagram of the action pathway of DMRTA2 in lung cancer cells.

To clarify the mechanism by which *DMRTA2* affects *p53*, we performed mass spectrometry analysis after overexpressing *DMRTA2* in A549 cells (Figure 11A), which identified HSP90β as one of the significantly interacting proteins. The literature reports indicate that HSP90β preferentially binds to wild-type *p53*, promoting its ubiquitin-mediated degradation and nuclear export. Knockdown of HSP90β resulted in the significantly inhibited proliferation of lung cancer cells in MTS and colony formation assays (Figure 11B,C), and Western blot analysis showed increased levels of *p53* and *p21* proteins (Figure 11D). Immunofluorescence and ubiquitination assays demonstrated reduced p53 ubiquitination and increased nuclear *p53* levels (Figure 11E,F). Co-immunoprecipitation experiments confirmed the interaction between *DMRTA2* and HSP90β (Figure 12A,B), and protein–protein interaction simulation results indicate that *DMRTA2* can form a stable complex with HSP90β (Figure 12C). Given that *DMRTA2* and HSP90β exert opposite effects on *p53* and that *DMRTA2* does not affect HSP90β expression, we hypothesized that *DMRTA2* inhibits the binding of HSP90β to *p53* by binding to HSP90β, thereby suppressing *p53* ubiquitination. MTS and colony formation assays showed that the overexpression of *DMRTA2* could reverse the pro-proliferative effect of HSP90β on lung cancer cells (Figure 12D,E), and Western blot results also restore the expression of *p53* and *p21* (Figure 12F). Finally, immunoprecipitation experiments with a gradient overexpression of *DMRTA2* indicated that as *DMRTA2* expression increased, the binding of HSP90β to p53 decreased (Figure 13A,B). In conclusion, *DMRTA2* activates the *p53* pathway by binding to HSP90β, inhibiting its ubiquitination and nuclear export of *p53*, and suppressing the proliferation and invasion of lung cancer cells (Figure 13C).

## 4. Discussion

Our study establishes *DMRTA2* as a context-dependent regulator in NSCLC with dual functionality dictated by *p53* status. While highly expressed in NSCLC and associated with better survival rates, *DMRTA2* demonstrates tumor-suppressive activity in wild-type *p53* cells by competitively binding HSP90β at its N-terminal ATP-binding and middle domains, thereby inhibiting HSP90β-p53 interaction. This molecular sequestration suppresses *p53* ubiquitination and nuclear export, leading to *p53/p21* pathway activation, cell cycle arrest, and the inhibition of proliferation/invasion (Figure 13C). The specificity of this mechanism is evidenced by *DMRTA2*’s exclusive interaction with HSP90β (not HSP90α) in *p53* wild-type environments, consistent with HSP90β’s role in stabilizing wild-type *p53* versus HSP90α’s preference for mutant *p53* [31,32].

Paradoxically, *DMRTA2* exhibits pro-tumorigenic properties in *p53*-mutant/null cells (e.g., SK-MES-1, H1299), promoting proliferation and invasion through two non-mutually exclusive mechanisms: First, potential complex formation with HSP90α may stabilize oncogenic mutant *p53*—a hypothesis supported by *DMRTA2*’s nucleolar localization (Figure 4B), which correlates with stress-responsive protein stabilization. Second, GSEA analysis reveals *DMRTA2*’s association with DNA damage repair pathways (nucleotide/base excision repair, mismatch repair, homologous recombination) and survival cascades (MAPK/NF-κB), suggesting alternative pathway activation when *p53* is compromised.

This functional duality is further illuminated by *DMRTA2*’s non-canonical characteristics: (1) Despite being a transcription factor, it minimally affects *p53* mRNA levels while significantly regulating *p21* expression; (2) Its nucleocytoplasmic shuttling, mediated by the interaction with nuclear transport protein IPO7, facilitates extranuclear functions; and (3) Multiple phosphorylation sites indicate post-translational regulation potential. Structural modeling reveals that *DMRTA2*-HSP90β binding occurs primarily in unstructured regions, with HSP90β’s critical functional domains being occupied, providing a mechanistic insight into how it disrupts HSP90β’s chaperone activity.

The clinical implications are twofold: First, *DMRTA2*’s association with DNA repair pathways suggests the possible modulation of radiotherapy/chemotherapy sensitivity. Second, its context-dependent functionality presents opportunities for *p53*-status-stratified therapies. Future studies should validate *DMRTA2*-HSP90α interactions in *p53*-mutant systems, characterize phosphorylation-dependent regulation, and explore its DNA damage response role—critical steps toward leveraging this multifaceted target for precision oncology.

## Data Availability

All the databases we used are openly available in TCGA at https://portal.gdc.cancer.gov/ (accessed on 6 April 2024), GEO at https://www.ncbi.nlm.nih.gov/geo/ (accessed on 6 April 2024), and BioGRID ORCs at https://orcs.thebiogrid.org/ (accessed on 6 April 2024).
